# Polypill Eligibility for Patients with Heart Failure with Reduced Ejection Fraction in the ASIAN-HF Registry: A Cross-Sectional Analysis

**DOI:** 10.5334/gh.1215

**Published:** 2023-06-15

**Authors:** Aishwarya Vijay, Wan Ting Tay, Tiew-Hwa K. Teng, Kanako Teramoto, Jasper Tromp, Wouter Ouwerkerk, Seet Yoong Lo, Wataru Shimizu, Mark D. Huffman, Carolyn S. P. Lam, Chanchal Chandramouli, Anubha Agarwal

**Affiliations:** 1 Department of Cardiology, Washington University in St. Louis, MO, USA; 2 National Heart Centre Singapore, Singapore; 3 School of Allied Health, University of Western Australia, WA, Australia; 4 Department of Cardiology, St. Marianna University School of Medicine, Kanagawa, Japan; 5 Department of Cardiology, University Medical Centre Groningen, Groningen, Netherlands; 6 Department of Clinical Epidemiology, Biostatistics, and Bioinformatics, Academic Medical Center, University of Amsterdam, Amsterdam, Netherlands; 7 Department of Cardiology, Tan Tock Seng Hospital, Singapore; 8 Department of Cardiovascular Medicine, Nippon Medical School, Tokyo, Japan; 9 Cardiovascular Division and Global Health Center, Washington University in St. Louis, MO, USA; 10 The George Institute for Global Health, University of New South Wales, Sydney, New South Wales, Australia

**Keywords:** polypill, HFrEF, global cardiovascular health, ASIAN-HF, guideline-directed medical therapy

## Abstract

**Background:**

The rates of guideline-directed medical therapy (GDMT) prescription for heart failure with reduced ejection fraction (HFrEF) in Asia remain sub-optimal. The primary objective of this study was to examine HFrEF polypill eligibility in the context of measured baseline prescription rates of individual components of GDMT among participants with HFrEF in Asia.

**Methods:**

A retrospective analysis of 4,868 patients with HFrEF from the multi-national ASIAN-HF registry was performed, and 3,716 patients were included in the final, complete case analysis. Eligibility for a HFrEF polypill, upon which patients were grouped and characterized, was based on the following: left ventricular systolic dysfunction (LVEF < 40% on baseline echocardiography), systolic blood pressure ≥ 100 mm Hg, heart rate ≥ 50 beats/minute, eGFR ≥ 30 mL/min/1.73 m, and serum potassium ≤ 5.0 mEq/L. Regression analyses were performed to evaluate associations of the baseline sociodemographic factors with HFrEF polypill eligibility.

**Results:**

Among 3,716 patients with HFrEF in the ASIAN-HF registry, 70.3% were eligible for a HFrEF polypill. HFrEF polypill eligibility was significantly higher than baseline rates of triple therapy prescription of GDMT across sex, all studied geographical regions, and income levels. Patients were more likely to be eligible for a HFrEF polypill if they were younger and male, with higher BMI and systolic blood pressure, and less likely to be eligible if they were from Japan and Thailand.

**Conclusion:**

The majority of patients with HFrEF in ASIAN-HF were eligible for a HFrEF polypill and were not receiving conventional triple therapy. HFrEF polypills may be a feasible and scalable implementation strategy to help close the treatment gap among patients with HFrEF in Asia.

## Introduction

The burden of heart failure with reduced ejection fraction (HFrEF) in Asia is increasing. Guideline-directed medical therapy (GDMT) is the cornerstone of treatment of patients with HFrEF and consists of beta-blockers (β-blockers), renin-angiotensin system inhibitors (RASi), mineralocorticoid receptor antagonists (MRA), and sodium glucose co-transporter 2 inhibitors (SGLT2i). A recent analysis demonstrated under-utilization of HFrEF GDMT (β-blockers, RASi, MRA) in 11 countries across Asia in the Asian Sudden Cardiac Death in Heart Failure (ASIAN-HF) registry [1].

The historical treatment algorithm for patients with HFrEF involves sequential initiation and titration of individual GDMT components over time. Previous work has suggested an additional reduction in mortality when GDMT components are used in combination compared with sequential initiation of these therapies [2]. As a result, the 2022 AHA/ACC/HFSA guideline for the management of heart failure recommends simultaneous initiation of GDMT, aligning with urgent recommendations by experts globally [3,4]. A HFrEF polypill is a fixed-dose combination of GDMT and a novel implementation strategy to simplify treatment and potentially improve GDMT adherence for untreated or undertreated patients with HFrEF [5]. The primary objective of this study is to examine HFrEF polypill eligibility in the context of measured baseline prescription rates of individual GDMT components among participants with HFrEF in the ASIAN-HF registry.

## Methods

A retrospective analysis of 4,868 patients with HFrEF (left ventricular ejection fraction (LVEF) < 40% on baseline echocardiography) from the multi-national ASIAN-HF registry was performed. Enrollment population as well as inclusion and exclusion criteria to the ASIAN-HF registry have been described previously [6]. Geographic blocs were grouped based on the United Nations Regional Groups. Regional income levels were categorized based on per capita income and corresponding World Bank classification [7]. Ethics approval was obtained from the local institutional review committee of each participating center, and all participants gave informed consent. The study conformed to the ethical guidelines in the Declaration of Helsinki.

A complete record of all medications was collected from each study participant at baseline. The following therapeutic classes of medications were included as GDMT for HFrEF based on guidelines during the recruitment period: β-blockers, ACEi/ARBs, and MRAs. Triple therapy was defined as being on all three therapeutic classes of medications. SGLT2i and ARNi data were not collected for this cohort, as data collection for the ASIAN-HF registry preceded the commercialization of SGLT2i and ARNi at study initiation.

## Statistical Methods

Eligibility for a HFrEF polypill was based on the following clinical and laboratory criteria for initiation of combination GDMT: left ventricular systolic dysfunction (LVEF < 40% on baseline echocardiography), systolic blood pressure ≥ 100 mm Hg, heart rate ≥ 50 beats/minute, eGFR ≥ 30 mL/min/1.73 m, and serum potassium ≤ 5.0 mEq/L [3,8,9]. Patients with HFrEF were grouped and characterized according to their eligibility for a HFrEF polypill. Differences between the two groups were tested with Student’s t-test, Wilcoxon rank-sum test, or the Chi square test for continuous and categorical variables, respectively.

Mixed effects logistic regression analyses were performed to evaluate the associations of the baseline sociodemographic factors with eligibility for a HFrEF polypill, including adjustments for age, sex, and body mass index. A random effect was also added to these models to account for within-study site clustering. Patients with incomplete data on the eligibility criteria for the HFrEF polypill were excluded from the analysis. STATA 14.0 (Stata Corp, College Station, TX, USA) was used for the statistical analyses.

## Results

**Figure 1:**
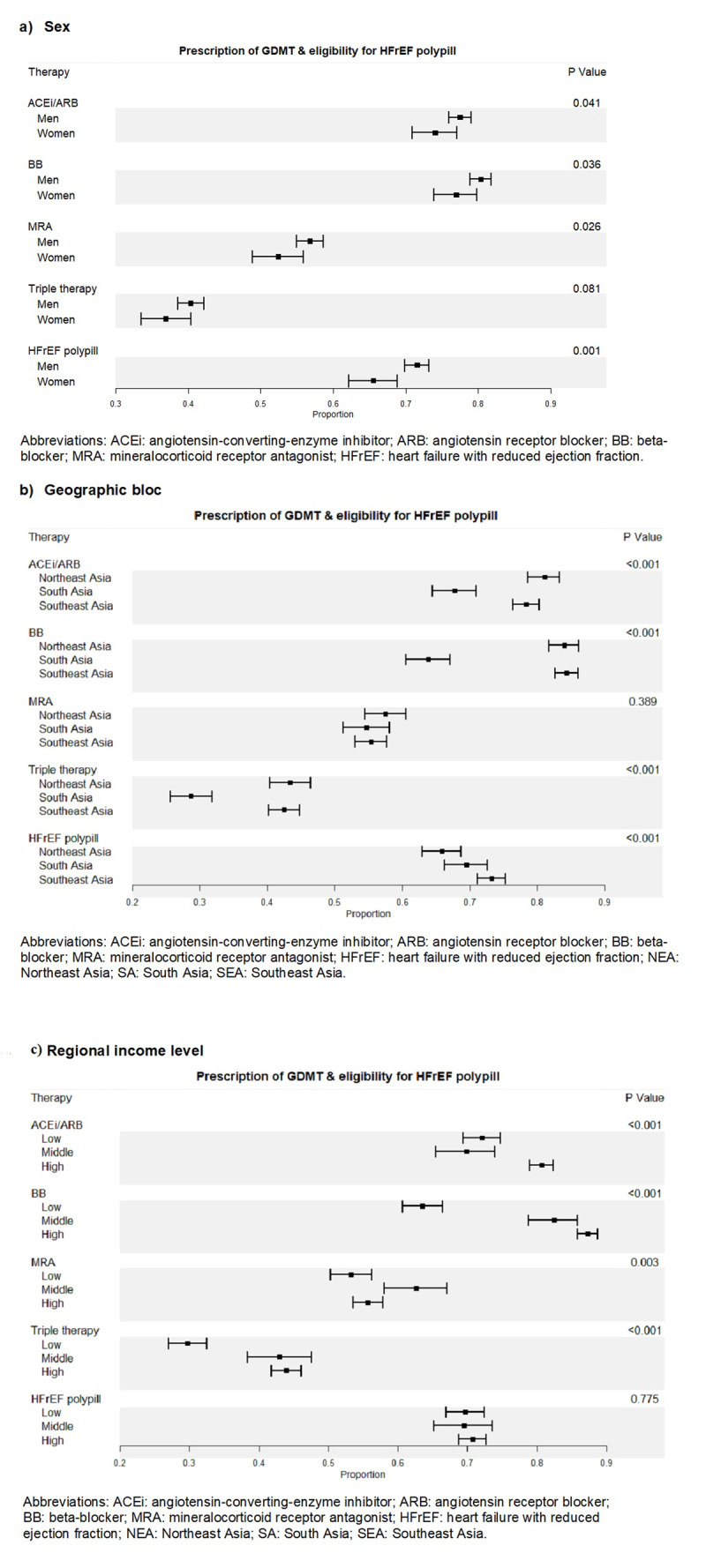
Prescription of individual and combined components of guideline directed medical therapy, triple therapy, and eligibility for HFrEF polypill by sex **(a)**, geographic bloc **(b)**, regional income level **(c)**.

The participant flowchart is shown in **Supplement: Figure 1**, and baseline characteristics and HFrEF polypill eligibility are reported in **Supplement: Table 1**. There were 4,868 patients with HFrEF in the ASIAN-HF registry; 1,152 patients were excluded due to data incompleteness. Among 3,716 patients with HFrEF in the ASIAN-HF registry, 70.3% (2,611/3,716) were eligible for a HFrEF polypill. More than 90% of patients were eligible based on heart rate ≥ 50 beats per minute and serum potassium < 5.0 mEq/L, while 88.7% (3,295/3,716) were eligible based on an eGFR ≥ 30 mL/min/1.73 m^2^, and 86.2% (3,205/3,716) were eligible based on a systolic blood pressure ≥ 100 mm Hg. Table 1 compares baseline characteristics of polypill eligible versus non-eligible HFrEF patients.

**Table 1:** Baseline characteristics of patients with HFrEF who were eligible or not eligible for HFrEF polypill therapy.


BASELINE CHARACTERISTICS	ELIGIBLE FOR HFrEF POLYPILL N = 2611	NOT ELIGIBLE FOR HFrEF POLYPILL N = 1105	p-VALUE

N (%)	2611 (70.3%)	1105 (29.7%)	

Age at baseline, years, mean (SD)	60.8 (13.0)	62.1 (13.1)	0.005

Men	2086 (79.9%)	829 (75.0%)	<0.001

NYHA class			<0.001

I	374 (16.0%)	98 (9.9%)	

II	1262 (54.1%)	534 (54.2%)	

III	600 (25.7%)	288 (29.2%)	

IV	97 (4.2%)	66 (6.7%)	

BMI, kg/m^2^	24.3 (21.8, 27.8)	23.6 (20.7, 26.5)	<0.001

Systolic BP, mmHg	120 (110, 131)	102 (92, 124)	<0.001

Diastolic BP, mmHg	72.0 (66, 80)	65 (58, 73)	<0.001

Heart rate, bpm	80.0 (70, 90)	75 (66, 86)	<0.001

LVEF at baseline, %	27 (21, 33)	27 (21, 33)	0.51

eGFR, mL/min/1.73 m^2^	66.5 (51.2, 84.6)	44.9 (24.1, 71.9)	<0.001

Potassium, mmol/L	4.1 (3.8, 4.5)	4.4 (4.0, 5.1)	<0.001

Etiology of HF			

Ischemic	1322 (50.7%)	580 (52.5%)	0.21

Non-Ischemic	1131 (43.3%)	474 (42.9%)	

Unknown	156 (6.0%)	51 (4.6%)	

Diabetes	1218 (46.6%)	542 (49.0%)	0.18

Coronary artery disease	1340 (51.3%)	626 (56.7%)	<0.01

Hypertension	1477 (56.6%)	600 (54.3%)	0.19

Atrial fibrillation/flutter	506 (19.4%)	240 (21.7%)	0.10

Peripheral arterial disease	96 (3.7%)	52 (4.7%)	0.14

ACE-inhibitor	1422 (54.9%)	488 (44.4%)	<0.001

ARB	804 (31.1%)	277 (25.2%)	<0.001

ACEi or ARB	2105 (81.3%)	726 (66.0%)	<0.001

Beta-blocker	2073 (80.1%)	863 (78.5%)	0.26

MRA	1513 (58.5%)	547 (49.7%)	<0.001

Geographical bloc			<0.001

Northeast Asia	712 (27.3%)	368 (33.3%)	

South Asia	587 (22.5%)	257 (23.3%)	

Southeast Asia	1312 (50.2%)	480 (43.4%)	

Ethnicity			<0.001

Chinese	714 (27.3%)	258 (23.3%)	

Indian	719 (27.5%)	299 (27.1%)	

Malay	470 (18.0%)	165 (14.9%)	

Japanese/Korean	523 (20.0%)	282 (25.5%)	

Thai/Filipino/Others	185 (7.1%)	101 (9.1%)	

Regional income level			0.78

Low	773 (29.6%)	336 (30.4%)	

Middle	333 (12.8%)	146 (13.2%)	

High	1505 (57.6%)	623 (56.4%)	


NYHA: New York Heart Association; BMI: body mass index; BP: blood pressure; LVEF: left ventricular ejection fraction; eGFR: estimated glomerular filtration rate; MDRD: modification of diet in renal disease; HF: heart failure; ACEi: angiotensin converting enzyme inhibitor; ARB: angiotensin receptor blocker; MRA: mineralocorticoid receptor antagonist.

Figure 1 shows that HFrEF polypill eligibility was significantly higher than baseline rates of triple therapy GDMT prescription across sex, all studied geographical regions, and income levels. HFrEF polypill eligibility was also higher than rates of individual ACEi, ARB, and BB adherence for the low-income level. **Supplement: Figure 2** shows the treatment ‘gap’ for polypill eligibility, defined as those who are eligible for HFrEF polypill but not receiving triple therapy. The proportion of patients in the treatment ‘gap’ was greatest in those from South Asia (46.56%; p < 0.001) or the lower income region (47.09%; p < 0.001) but not different between sexes (male 41.05% versus female 38.79%; p = 0.251).

Table 2 shows the comparison of characteristics among individuals who were and were not eligible for a HFrEF polypill. Patients who were eligible for a HFrEF polypill were younger (age 60.8 years ± 13.0 vs. 62.1 ± 13.1, p = 0.005), more likely to be male (79.9% vs. 75.0%), had a higher BMI (24.3 [21.8, 27.8] kg/m^2^ vs. 23.6 [20.7, 26.5] kg/m^2^), had a higher blood pressure (median [IQR] systolic and diastolic blood pressure (120/72 [110/66, 131/80] mmHg vs. 102/65 [92/58, 124/73] mmHg), and were more likely to be from Southeast Asia compared with all other regions (50.2% vs. 43.4%). Baseline ACEi, ARB or MRA use was each associated with higher HFrEF polypill eligibility (ACEi 54.9% vs. 44.4%, ARB 31.1% vs. 25.2% and MRA 58.5% versus 49.7%).

**Table 2:** Association of sociodemographic and geographic factors with HFrEF polypill eligibility.


	N	ELIGIBLE FOR HFrEF POLYPILL	UNADJUSTED OR (95% CI)	p-VALUE	ADJUSTED* OR (95% CI)	p-VALUE

**Sex**						

Men	2915	2086 (71.6%)	1.00 (Ref)		1.00 (Ref)	

Women	801	525 (65.5%)	0.76 (0.64, 0.89)	0.001	0.77 (0.65, 0.91)	<0.01

**Geographical bloc**						

Northeast Asia	1080	712 (65.9%)	0.85 (0.70, 1.03)	0.09	0.82 (0.61, 1.09)	0.17

South Asia	844	587 (69.6%)	1.00 (Ref)		1.00 (Ref)	

Southeast Asia	1792	1312 (73.2%)	1.20 (1.00, 1.43)	0.05	1.01 (0.70, 1.46)	0.97

**Regional income level**						

Low	1109	773 (69.7%)	0.95 (0.81, 1.12)	0.55	1.05 (0.77, 1.44)	0.74

Middle	479	333 (69.5%)	0.94 (0.76, 1.17)	0.60	0.99 (0.68, 1.43)	0.94

High	2128	1505 (70.7%)	1.00 (Ref)		1.00 (Ref)	

**Region**						

Hong Kong	50	35 (70.0%)	1.02 (0.55, 1.90)	0.95	0.95 (0.43, 2.14)	0.91

India	844	587 (69.6%)	1.00 (Ref)		1.00 (Ref)	

Indonesia	213	148 (69.5%)	1.00 (0.72, 1.38)	0.99	0.88 (0.54, 1.43)	0.61

Japan	538	328 (61.0%)	0.68 (0.54, 0.86)	0.001	0.64 (0.43, 0.93)	0.02

Korea	268	196 (73.1%)	1.19 (0.88, 1.62)	0.26	1.11 (0.72, 1.71)	0.65

Malaysia	291	221 (76.0%)	1.38 (1.02, 1.88)	0.04	1.23 (0.79, 1.92)	0.36

Philippines	52	38 (73.1%)	1.19 (0.63, 2.23)	0.59	1.00 (0.48, 2.11)	0.99

Singapore	1,048	793 (75.7%)	1.36 (1.11, 1.67)	<0.01	1.17 (0.82, 1.68)	0.38

Taiwan	224	153 (68.3%)	0.94 (0.69, 1.30)	0.72	0.86 (0.55, 1.35)	0.51

Thailand	188	112 (59.6%)	0.65 (0.47, 0.89)	<0.01	0.59 (0.36, 0.97)	0.04


* Adjusted for age and sex, and site-level clustering using a random effect.

Compared to patients from India, patients from Singapore were more likely to be eligible for a HFrEF polypill (unadjusted OR 1.36 [95% CI 1.11–1.67], p < 0.01; Table 2), but the association was attenuated after adjustment for age, sex, and site-level clustering (adjusted OR 1.17 [95% CI 0.82–1.68], p = 0.38). Compared to patients from India, patients from Japan (adjusted OR = 0.64 [95% CI 0.43–0.93], p = 0.02) and Thailand (adjusted OR = 0.59 [95% CI 0.36–0.97], p = 0.04) were less likely to be eligible for a HFrEF polypill, primarily due to having SBP < 100 for patients from Thailand, a higher proportion had eGFR < 30 as compared to patients from India.

## Discussion

In this study of patients with HFrEF from 10 different regions across Asia, 70.3% of participants were eligible for a HFrEF polypill. HFrEF polypill eligibility was significantly higher than baseline rates of triple therapy GDMT prescription across sex, all studied geographical regions, and income levels. The treatment ‘gap,’ which may indicate populations most likely to benefit from a polypill, was greatest in those from India or the lower income regions. These findings support previous research demonstrating more than 80% of patients with HFrEF in India were eligible for a HFrEF polypill [9].

This study found that patients were more likely to be eligible for a HFrEF polypill if they were younger and male and less likely to be eligible if they were from Japan and Thailand. This may be due to the demonstrated differential drug clearance as well as differential baseline SBP based on sex, age, and ethnicity, which has been previously demonstrated [10]. Key considerations, as HFrEF polypills are implemented in target populations, include availability of multiple HFrEF polypill doses to prioritize safety and tolerability through low-dose initiation with subsequent titration to higher dose HFrEF polypills, and potential customization of dose combinations [5].

Limitations of this analysis include that laboratory data used to determine HFrEF polypill eligibility were obtained at the time of hospital admission, and that renal function frequently changes during hospitalization in many HFrEF patients, which may influence HfrEF polypill eligibility. Key components of GDMT, including SGLT2i and ARNi compounds, were not included in this analysis due to limited data availability in the ASIAN-HF registry. Finally, more than 1,000 people were excluded from the analysis due to data completeness; however, it is uncertain how this would influence the estimates provided.

## Conclusion

Polypills may improve adherence by up to 44% as compared to a multi-drug regimen [11]. This study indicated that 7 out of every 10 patients were eligible for HFrEF polypill therapy in a representative cohort of patients with HFrEF across Asia. Given sub-optimal rates of GDMT prescription and potential adherence in Asia, HFrEF polypills may be a feasible and scalable implementation strategy to help to close the treatment gap among HFrEF patients in undertreated populations.

## Data Accessibility Statement

Data is available upon request from the corresponding author and the ASIAN-HF committee.

## Additional File

The additional file for this article can be found as follows:

10.5334/gh.1215.s1 Supplemental Table and Figures. Supplemental Table 1 and Figures 1 to 2.
