# Supplementary material for: Polypill Eligibility for Patients with Heart Failure with Reduced Ejection Fraction in the ASIAN-HF Registry: A Cross-Sectional Analysis
Source: Glob Heart. 2023 Jun 15;18(1):33. doi: 10.5334/gh.1215 (PMC10275129; doi:10.5334/gh.1215)
Supplement: Supplemental Table and Figures. — Supplemental Table 1 and Figures 1 to 2. [file gh-18-1-1215-s1.pdf]

Polypill Eligibility for Patients with Heart Failure with Reduced Ejection Fraction in the ASIAN-HF Registry: A Cross-Sectional Analysis

Supplement

Supplement: Table 1. Assessment of eligibility criteria for HFrEF polypill.

Individual criteria	Criteria met, n (%)
Complete data	3716
LVEF < 40%	3716 (100%)
Systolic BP >= 100 mmHg	3205 (86.2%)
Heart rate >= 50 beats/minute	3684 (99.1%)
eGFR >= 30 mL/min/1.73m^2	3295 (88.7%)
Serum potassium <= 5.0 mEq/L	3436 (92.5%)
**Overall combined criteria**	**2611 (70.3%)**

LVEF: left ventricular ejection fraction; BP: blood pressure; eGFR: estimated glomerular filtration rate

Supplement: Figure 1. Participant flow diagram.

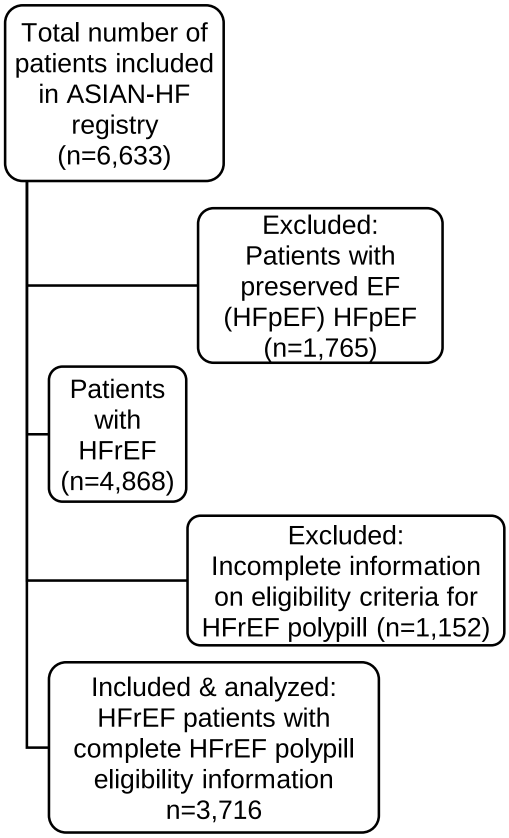

Supplement: Figure 1 Legend. This figure is a STROBE (Strengthening the Reporting of Observational Studies in Epidemiology) diagram of patient recruitment displaying the progress through stages of analysis.

Supplement: Figure 2. Treatment gap demonstrating proportion of participants eligible for a HFrEF polypill and not on triple therapy at baseline.

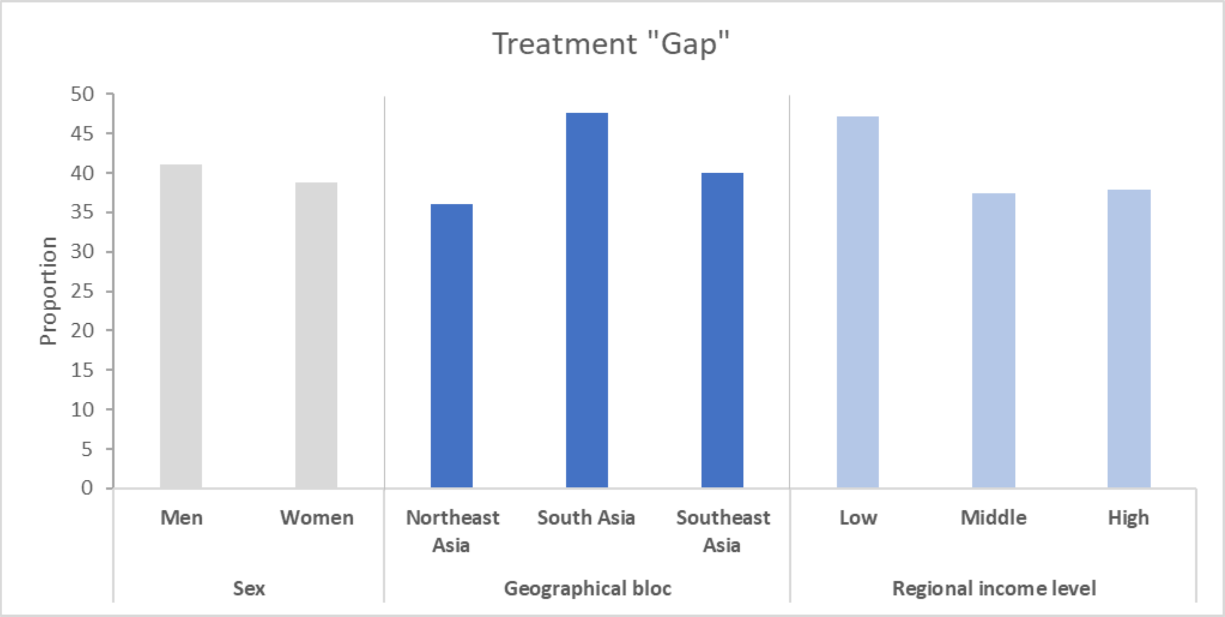

Supplement: Figure 2 Legend. Proportions displayed on y-axis indicate the percentage of the select population that were eligible for a HFrEF polypill and not on triple therapy at baseline.
